# Fundamental social motives measured across forty-two cultures in two waves

**DOI:** 10.1038/s41597-022-01579-w

**Published:** 2022-08-16

**Authors:** Cari M. Pick, Ahra Ko, Douglas T. Kenrick, Adi Wiezel, Alexandra S. Wormley, Edmond Awad, Laith Al-Shawaf, Oumar Barry, Yoella Bereby-Meyer, Watcharaporn Boonyasiriwat, Eduard Brandstätter, Suzan Ceylan-Batur, Bryan K. C. Choy, Ana Carla Crispim, Julio Eduardo Cruz, Daniel David, Oana A. David, Renata Pereira Defelipe, Pinar Elmas, Agustín Espinosa, Ana Maria Fernandez, Velichko H. Fetvadjiev, Stefka Fetvadjieva, Ronald Fischer, Silvia Galdi, Oscar Javier Galindo-Caballero, Elena V. Golovina, Galina M. Golovina, Luis Gomez-Jacinto, Sylvie Graf, Igor Grossmann, Pelin Gul, Peter Halama, Takeshi Hamamura, Shihui Han, Lina S. Hansson, Hidefumi Hitokoto, Martina Hřebíčková, Darinka Ilic, Jennifer Lee Johnson, Mane Kara-Yakoubian, Johannes A. Karl, Jinseok P. Kim, Michal Kohút, Julie Lasselin, Hwaryung Lee, Norman P. Li, Anthonieta Looman Mafra, Oksana Malanchuk, Simone Moran, Asuka Murata, Jinkyung Na, Serigne Abdou Lahat Ndiaye, Jiaqing O, Ike E. Onyishi, Eddieson Pasay-an, Muhammed Rizwan, Eric Roth, Sergio Salgado, Elena S. Samoylenko, Tatyana N. Savchenko, Catarina Sette, A. Timur Sevincer, Eric Skoog, Adrian Stanciu, Eunkook M. Suh, Daniel Sznycer, Thomas Talhelm, Fabian O. Ugwu, Ayse K. Uskul, Irem Uz, Jaroslava Varella Valentova, Marco Antonio Correa Varella, Liuqing Wei, Danilo Zambrano, Michael E. W. Varnum

**Affiliations:** 1 Department of Psychology, Arizona State University, Tempe, AZ 85287 USA; 2 Office of the Chief Scientist, Environmental Defense Fund, New York, NY 10010 USA; 3 Department of Economics, University of Exeter Business School, Exeter EX4 4PU, England, UK; 4 Department of Psychology, University of Colorado, Colorado Springs, CO 80309 USA; 5 Department of Psychology, University Cheikh Anta Diop of Dakar (UCAD), Dakar, 10700 Senegal; 6 Department of Psychology, Ben-Gurion University of the Negev, Beer-Sheva, 84105 Israel; 7 Faculty of Psychology, Chulalongkorn University, Bangkok, 10330 Thailand; 8 Department of Economic Psychology, Johannes Kepler University Linz, 4040 Linz, Austria; 9 Department of Psychology, TOBB University of Economics and Technology, 06510 Ankara, Turkey; 10 School of Social Sciences, Singapore Management University, Singapore, 188065 Singapore; 11 eduLab21, Ayrton Senna Institute, São Paulo, 05423-040 Brazil; 12 Department of Psychology, Universidad de los Andes, Bogotá, Cundinamarca Colombia; 13 Department of Clinical Psychology and Psychotherapy, Babeş-Bolyai University, Cluj-Napoca, 400347 Romania; 14 Department of Experimental Psychology, Institute of Psychology, University of São Paulo, São Paulo, 05508-030 Brazil; 15 Department of Psychology, Adnan Menderes University, 09010 Aydın, Turkey; 16 Grupo de Psicología Política y Social (GPPS), Departamento de Psicología, Pontificia Universidad Católica del Perú, San Miguel, 15088 Lima, Peru; 17 School of Psychology, University of Santiago, Santiago, Estación Central, Región Metropolitana Chile; 18 Department of Psychology, University of Amsterdam, 1018 WS Amsterdam, Netherlands; 19 WorkWell Research Unit, North-West University, Potchefstroom, 2520 South Africa; 20 Department of Bulgarian Language, Sofia University, Sofia, Bulgaria; 21 School of Psychology, Victoria University of Wellington, Wellington, 6012 New Zealand; 22 Instituto D’Or de Pesquisa e Ensino, Rio de Janeiro, 22281-100 Brazil; 23 Department of Psychology, University of Campania Luigi Vanvitelli, 81100 Caserta, Italy; 24 Faculty of Education, Human and Social Sciences, Universidad Manuela Beltran, Bogotá, Colombia; 25 Institute of Psychology Russian Academy of Science, Moscow, 129366 Russia; 26 Department of Social Psychology, Social Work and Social Anthropology, University of Málaga, 29016 Málaga, Spain; 27 Institute of Psychology, Czech Academy of Sciences, 110 00 Nové Město, Prague, Czechia; 28 Department of Psychology, University of Waterloo, Waterloo, Ontario N2L 3G1 Canada; 29 Department of Sustainable Health (Campus Fryslân), University of Groningen, 8911CE Leeuwarden, Netherlands; 30 Center of Social and Psychological Sciences, Slovak Academy of Sciences, 841 04, Bratislava, Slovakia; 31 School of Psychology, Curtin University, Bentley, WA 6102 Perth, Australia; 32 School of Psychological and Cognitive Sciences, Peking University, Beijing, 100871 China; 33 Stress Research Institute, Department of Psychology, Stockholm University, 106 91 Stockholm, Sweden; 34 Division of Psychology, Department of Clinical Neuroscience, Karolinska Institutet, 171 77 Solna, Sweden; 35 Osher Center for Integrative Medicine, ME Neuroradiologi, Karolinska Universitetssjukhuset, 171 77 Solna, Sweden; 36 School & Graduate School of Humanities, Kwansei Gakuin University, Nishinomiya, Hyogo 662-8501 Japan; 37 Department of Psychology, Faculty of Philosophy, University of Niš, Niš, 18000 Serbia; 38 Department of Community Sustainability, Michigan State University, East Lansing, MI 48824 USA; 39 Department of Psychology, Toronto Metropolitan University, Toronto, Ontario M5B 2K3 Canada; 40 School of Psychology, Dublin City University, Dublin, 9 Ireland; 41 Department of Psychology, Yonsei University, Seoul, 03722 South Korea; 42 Faculty of Philosophy and Arts, University of Trnava, 917 01 Trnava, Slovakia; 43 Institute for Social Research, University of Michigan, Ann Arbor, MI 48104 USA; 44 Department of Management, Ben-Gurion University of the Negev, Beer-Sheva, 84105 Israel; 45 Graduate School of Letters, Hokkaido University, Sapporo, Hokkaido 060-0810 Japan; 46 Department of Psychology, Sogang University, Seoul, 04107 South Korea; 47 Department of Sociology, University Cheikh Anta Diop of Dakar (UCAD), Dakar, 10700 Senegal; 48 Department of Psychology, Aberystwyth University, Aberystwyth, SY23 3UX Wales UK; 49 Department of Psychology, University of Nigeria, Nsukka, Nigeria; 50 College of Nursing, University of Hail, Hail, 55476 Saudi Arabia; 51 Department of Psychology, University of Haripur, Haripur, 22620 Khyber Pakhtunkhwa Pakistan; 52 Experimental Research Unit (ERU), Department of Psychology, Universidad Católica Boliviana, La Paz, Bolivia; 53 Department of Management and Economics, Universidad de La Frontera, Temuco, Araucanía Chile; 54 Department of Psychology, University of Hamburg, 20146 Hamburg, Germany; 55 Department of Peace and Conflict Research, Uppsala University, 753 20 Uppsala, Sweden; 56 Department of Monitoring Society and Social Change, Gesis-Leibniz Institute for the Social Sciences, 68072 Mannheim, Germany; 57 Department of Psychology, Oklahoma State University, Stillwater, OK 74078 USA; 58 Behavioral Science, University of Chicago, Chicago, IL 60637 USA; 59 Department of Psychology, Alex Ekwueme Federal University, Ndufu-Alike, Ebonyi State Nigeria; 60 School of Psychology, University of Kent, Canterbury, CT2 7NP UK; 61 Department of Education, Hubei University, Wuhan, Hubei 430061 China; 62 Department of Psychology, Fundación Universitaria Konrad Lorenz, Bogotá, Colombia

**Keywords:** Human behaviour, Anthropology

## Abstract

H﻿ow does psychology vary across human societies? The fundamental social motives framework adopts an evolutionary approach to capture the broad range of human social goals within a taxonomy of ancestrally recurring threats and opportunities. These motives—self-protection, disease avoidance, affiliation, status, mate acquisition, mate retention, and kin care—are high in fitness relevance and everyday salience, yet understudied cross-culturally. Here, we gathered data on these motives in 42 countries (*N* = 15,915) in two cross-sectional waves, including 19 countries (*N* = 10,907) for which data were gathered in both waves. Wave 1 was collected from mid-2016 through late 2019 (32 countries, *N* = 8,998; 3,302 male, 5,585 female; *M*_*age*_ = 24.43, *SD* = 7.91). Wave 2 was collected from April through November 2020, during the COVID-19 pandemic (29 countries, *N* = 6,917; 2,249 male, 4,218 female; *M*_*age*_ = 28.59, *SD* = 11.31). These data can be used to assess differences and similarities in people’s fundamental social motives both across and within cultures, at different time points, and in relation to other commonly studied cultural indicators and outcomes.

## Abstract (summary)

**Table:** 

Measurement(s)	Motivation • Emotional Well-being • Socioeconomic Indicator • Culture • Cultural Diversity
Technology Type(s)	survey method • digital curation
Sample Characteristic - Organism	Homo sapiens
Sample Characteristic - Location	Australia • Austria • Bolivia • Brazil • Bulgaria • Canada • Chile • China • Colombia • Czech Republic • Germany • Hong Kong • India • Israel • Italy • Japan • Kenya • Lebanon • Mexico • The Netherlands • New Zealand • Nigeria • Pakistan • Peru • The Philippines • Portuguese Republic • Romania • Russia • Saudi Arabia • Senegal • Serbia • Singapore • Slovak Republic • South Korea • Spain • Sweden • Thailand • Turkey • Uganda • Ukraine • United Kingdom • United States of America

## Background & Summary

As human beings have come into increasing contact with people from other parts of the globe, understanding the psychological differences and similarities between people of different cultures has become increasingly critical^1–3^, with broad-reaching economic and political implications. Over the last few decades, researchers in fields including anthropology, evolutionary biology, and cognitive science have investigated questions about universals in human nature^4–8^. During the same period, there has been increasing interest in psychological differences across cultures^2,9–11^. These approaches are, of course, complementary^12,13^. We suggest a new way of thinking about cultural variation, in terms of a set of fundamental motivational systems evolved to deal with the universal problems and opportunities that human beings have regularly confronted in their social relationships—involving self-protection, disease avoidance, affiliation, status, mate acquisition, mate retention, and kin care (see Table 1 for a brief description of each motive and sample items from the Fundamental Social Motives Inventory^14^). In the face of these recurring challenges and opportunities, humans are presumed to have evolved a set of fundamental social motives—systems of perception, cognition, and affect that direct behavior in ways that help address these challenges^15,16^.

**Table 1:** Brief descriptions of each of the seven fundamental social motives, and two sample items from each of the 11 six-item subscales of the Fundamental Social Motives Inventory (FSMI).

Fundamental Social Motives			Subscale Sample Items
	Self-Protection	Archeological and anthropological studies of ancestral societies suggest homicide and assault rates many times greater than those found in modern societies^36,37^.	• I think about how to protect myself from dangerous people
			• I am motivated to protect myself from dangerous others.
	Disease Avoidance	Ancestrally, contagious illnesses were responsible for the deaths of a substantial portion of infants and for a substantial number of deaths among adults, as well^37^. Increased population density after the onset of agriculture exacerbated this problem^38^.	• I avoid places and people that might carry diseases.
			• When someone near me is sick, it doesn’t bother me very much. (R)
	Affiliation	Anthropological evidence suggests that individuals living under ancestral conditions would not have produced sufficient calories to feed themselves or their offspring without the existence of cooperative risk-pooling alliances^39^.	*Group subscale*
			• I enjoy working with a group to accomplish a goal.
			• Getting along with the people around me is a high priority.
			*Independence subscale*
			• Being apart from my friends for long periods of time does not bother me.
			• Having time alone is extremely important to me.
			*Exclusion Concern subscale*
			• I would be extremely hurt if a friend excluded me.
			• It bothers me when groups of people I know do things without me.
	Status	Individuals achieving positions of respect in ancestral groups likely had increased access to resources and desirable mates^40^.	• It’s important to me that others respect my rank or position.
			• I do things to ensure that I don’t lose the status I have.
	Mate Acquisition	All ancestors of currently existing sexually reproducing organisms, including *Homo sapiens*, were successful in attracting at least one mate.	• I spend a lot of time thinking about ways to meet possible dating partners.
			• I am interested in finding a new romantic/sexual partner.
	Mate Retention	Because humans are altricial, our helpless offspring benefit greatly from resources and care provided by two parents^41^.	*Mate Retention (General) subscale*
			• It is important to me that my partner is sexually loyal to me.
			• It would not be that big a deal to me if my partner and I broke up. (R)
			*Breakup Concern subscale*
			• I often think about whether my partner will leave me.
			• I worry about others stealing my romantic/sexual partner.
	Kin Care	Beyond caring for their direct descendants, human beings also traditionally shared essential resources and protection within wider kin groups^39^. Humans are a relatively slow life history species^42^ and human psychology is shaped by inclusive fitness^43^.	*Family subscale*
			• Caring for family members is important to me.
			• It is extremely important to me to have good relationships with my family members.
			*Children subscale*
			• I often think about how I could stop bad things from happening to my children.
			• Providing for my children is important to me.

The FSMI includes multiple subscales of Affiliation (i.e., Group, Independence, and Exclusion Concern), Mate Retention (i.e., General and Breakup Concern), and Kin Care (i.e., Family and Children).

This framework has generated a number of interesting findings. Overall, these studies have found that cognitive processes, affect, and behaviors vary, in adaptively functional ways, as different fundamental social motives are activated. Activating Self-Protection versus Mate Seeking versus Disease Avoidance concerns, for example, has very different, yet functionally sensible, effects on attention^17,18^, perception of others’ emotions^19^, conformity^20^, economic decision-making^21,22^, aggression^23^, responses to persuasion^24^, and detection of threat-cues in potential enemies versus allies^25^. Activation of parenting (Kin Care) motives has also been linked to a number of functionally sensible outcomes^26^.

Furthermore, the fundamental social motives are linked to individual differences in functionally relevant ways. For example, consistent with principles of differential parental investment and sexual selection, which have been linked to male competition for more selective female mating partners across species, Mate Seeking leads to more risk-taking behaviors in men, but more conforming and group-oriented behavior in women^20,21^. Other research finds that chronic activation of motives (e.g., Status, Mate Seeking, Self-Protection) links in sensible ways to life-history-relevant demographic variables, such as one’s sex, age, and number of children^14,27^. In addition, at the individual level, fundamental social motives appear sensibly correlated with personality traits such as the Big Five factors. For example, agreeableness is correlated with the motive to affiliate with groups, and neuroticism is correlated with the Self-Protection motive. At the same time, the motives demonstrate discriminant validity from these and other dimensions^14^. Thus, fundamental social motives affect a wide range of social, cognitive, affective, and behavioral processes and are systematically linked to demographic and individual differences.

Might the fundamental social motives also vary systematically across human societies? Although these dimensions are high in both fitness relevance and everyday salience, they have been largely missing from the study of human cultural variation. How might these fundamental social motives map onto previously studied dimensions of cultural differences? How might the picture of human cultural variation look if we took these motives into account? How might a consideration of fundamental social motives influence our understanding of cultural similarity or distance among the world’s societies? We have not yet found existing data capable of answering questions such as these. Here, therefore, a team of international collaborators gathered data on the fundamental social motives across 42 societies in two waves.

## Methods

This research was approved by the Institutional Review Board (IRB) at Arizona State University (ASU).

### Participants

**Fig. 1:**
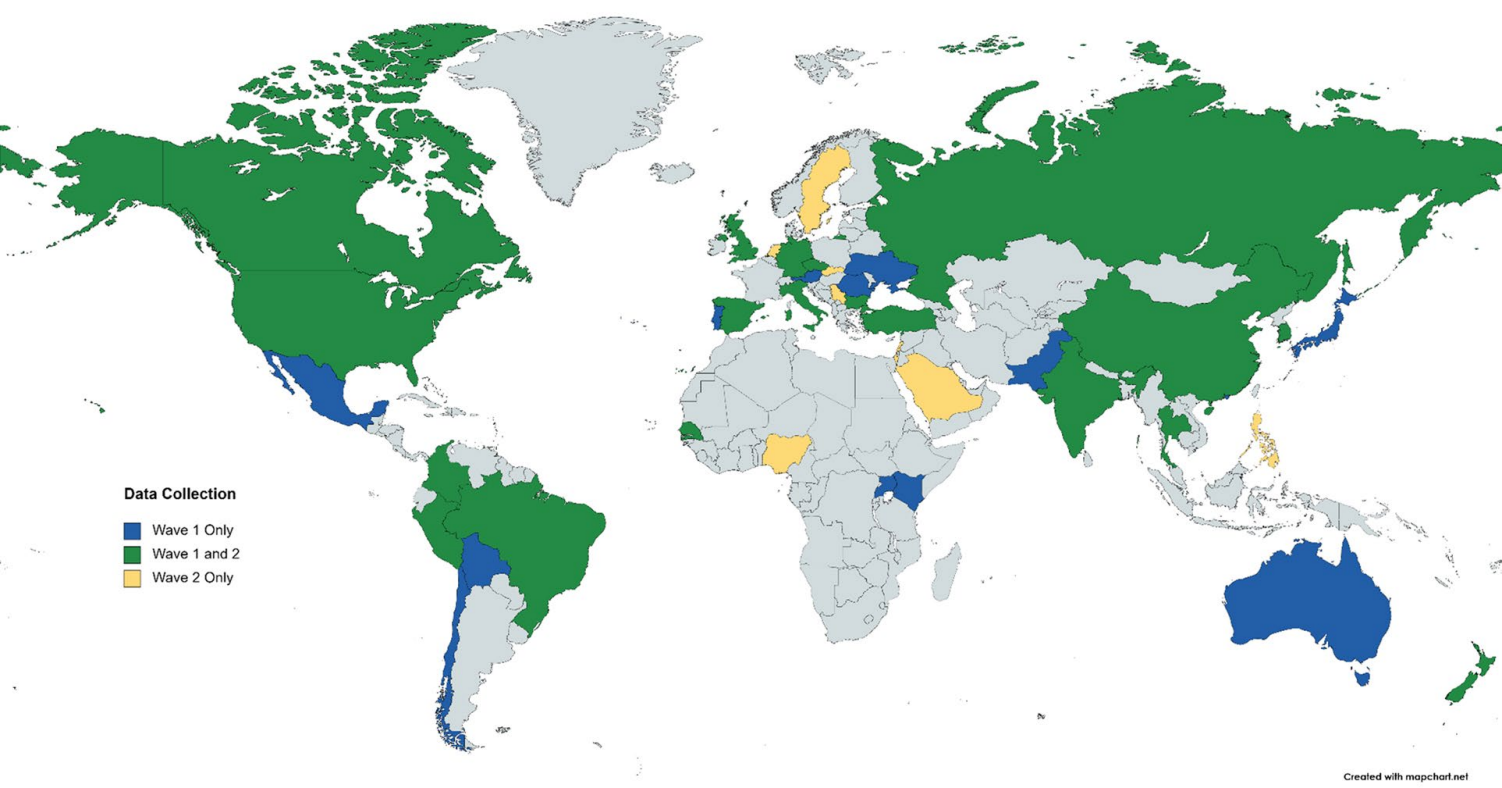
Countries in which data were collected in Waves 1 and 2. Countries in which data were collected only during Wave 1 are indicated in blue (*n* = 13), data collection only in Wave 2 is indicated in yellow (*n* = 10), and data collection in both Waves 1 and 2 is indicated in green (*n* = 19).

Data were gathered in two waves, from a total of 42 countries (total *N = *15,915). Nineteen countries were represented in both waves (*N = *10,907) (see Fig. 1).

The first wave of data was collected from mid-2016 through late 2019 in 32 countries from all inhabited continents (Supplementary Table 1 provides data collection timeframes by country; Table 2 provides full list of countries in each wave). Data were collected assessing fundamental social motives of 8,998 individuals (3,302 male, 5,585 female, 111 “other” or declined to answer; *M*_age_ = 24.43, *SD*_age_ = 7.91, *min*_age_ = 18). Supplementary Table 2 provides detailed demographic information by country.

**Table 2:** Data were collected in a total of 42 countries across two waves.

Country/Society	Wave 1 Data Collection	Wave 2 Data Collection
*Australia*	X	
*Austria*	X	
*Bolivia*	X	
*Brazil*	X	X
*Bulgaria*	X	X
*Canada*	X	X
*Chile*	X	
*China (Mainland)*	X	X
*Colombia*	X	X
*Czech Republic*	X	X
*Germany*	X	X
*Hong Kong*	X	
*India*	X	X
*Israel*		X
*Italy*	X	X
*Japan*	X	
*Kenya*	X	
*Lebanon*		X
*Mexico*	X	
*Netherlands*		X
*New Zealand*	X	X
*Nigeria*		X
*Pakistan*	X	
*Peru*	X	X
*Philippines*		X
*Portugal*	X	
*Romania*	X	
*Russia*	X	X
*Saudi Arabia*		X
*Senegal*	X	X
*Serbia*		X
*Singapore*		X
*Slovakia*		X
*South Korea*	X	X
*Spain*	X	X
*Sweden*		X
*Thailand*	X	X
*Turkey*	X	X
*Uganda*	X	
*Ukraine*	X	
*United Kingdom*	X	X
*United States*	X	X

Wave 1 data were collected in 32 countries. Wave 2 data were collected in 29 countries. Nineteen countries were represented in both Waves.

The second wave of data was collected from April 2020 through November 2020, during the first year of the COVID-19 global pandemic, in 29 countries (Supplementary Table 1 provides data collection timeframes by country; Table 2 provides full list of countries in each wave). Data were collected assessing fundamental social motives of 6,917 individuals (2,249 male, 4,218 female, 450 “other” or declined to answer; *M*_age_ = 28.59, *SD*_age_ = 11.31, *min*_age_ = 18). Supplementary Table 2 provides detailed demographic information by country.

Data were collected via convenience sampling, including from university populations, community samples, and paid online workforces (e.g., Prolific, Amazon’s Mechanical Turk). Supplementary Table 1 provides sample type details by country, as well as city or region of data collection, if applicable. The target sample size was 200 participants per country, but this target was not reached in some countries due to limitations in data collection. Wave 1 sample *N*s ranged from 84 participants (Russia) to 769 participants (Senegal). Wave 2 sample *N*s ranged from 67 participants (Serbia) to 612 participants (Senegal). Supplementary Table 2 provides sample size by country in each wave. In some countries, multiple teams of researchers collected data during the same wave (see Supplementary Table 1 for details on countries with “subsamples”). Surveys were administered either via paper-and-pencil or computer/tablet.

### Procedures

Parallel survey procedures were used in Waves 1 and 2. For societies in which English is not primarily spoken, collaborators collecting the data translated the survey materials into local languages. Supplementary Table 1 provides survey language and translation procedure details by country.

#### Fundamental Social Motives Inventory

After providing informed consent, participants completed the Fundamental Social Motives Inventory (FSMI)^14^, a 66-item instrument assessing 11 motive subdimensions: Self-Protection, Disease Avoidance, Affiliation (Exclusion Concern), Affiliation (Group), Affiliation (Independence), Status, Mate Seeking, Breakup Concern, Mate Retention, Kin Care (Family), and Kin Care (Children). Participants who did not have children were instructed not to complete the Kin Care (Children) items, and those not in romantic relationships were instructed not to complete the Breakup Concern and Mate Retention items. In some samples, participants indicated their relationship status and whether they had children before completing the FSMI, and they were subsequently not shown Kin Care (Children) or Breakup Concern and Mate Retention items if they did not have children or a relationship, respectively. For means and standard deviations of fundamental social motives by country in Wave 1 see Table 3, and for Wave 2 see Table 4.

**Table 3:** Fundamental Social Motive means (and standard deviations) by country in Wave 1.

Country/Society	SPO	DIS	AFG	AFI	AFX	STA	MAT	MRB	MRT	KCF	KCC
*Australia*	4.20(1.19)	3.65(1.17)	4.73(0.97)	4.53(1.11)	4.62(1.27)	4.41(0.97)	3.25(1.58)	3.14(1.79)	5.80(1.00)	5.57(1.33)	4.89(1.57)
*Austria*	3.64(1.27)	3.40(1.09)	5.31(0.97)	3.74(1.16)	4.20(1.04)	4.38(1.02)	3.13(1.54)	2.70(1.55)	6.03(0.84)	5.36(1.21)	5.75(1.21)
*Bulgaria*	4.69(1.33)	3.97(1.19)	4.94(1.04)	4.09(1.20)	4.08(1.28)	4.64(1.18)	2.92(1.50)	3.21(1.57)	6.22(0.76)	5.76(1.07)	6.01(1.50)
*Bolivia*	4.86(1.02)	3.82(1.09)	4.73(1.07)	4.77(1.11)	3.88(1.42)	4.66(1.09)	3.49(1.29)	4.08(1.60)	5.67(0.84)	5.28(1.27)	6.21(0.82)^†^
*Brazil*	4.78(1.31)	3.55(1.16)	5.02(1.08)	4.73(1.22)	4.28(1.50)	4.27(1.19)	3.20(1.60)	2.94(1.43)	5.74(0.94)	5.33(1.28)	6.00(1.14)
*Canada*	4.19(1.07)	3.98(1.06)	4.57(0.93)	4.14(1.07)	4.47(1.22)	4.40(0.94)	3.77(1.31)	3.28(1.48)	5.44(1.09)	5.62(1.14)	4.07(1.18)
*Chile*	4.41(1.27)	3.51(1.23)	5.03(1.02)	4.53(1.14)	4.17(1.23)	3.91(1.22)	3.21(1.41)	3.58(1.58)	5.49(1.02)	5.57(1.23)	5.51(1.69)
*China*	5.15(0.84)	4.54(1.00)	5.12(0.85)	4.59(1.13)	5.26(0.94)	5.08(0.87)	3.79(1.25)	4.29(1.48)	5.64(0.96)	5.90(1.03)	5.15(1.37)
*Colombia*	4.42(1.23)	3.56(1.25)	4.93(1.04)	4.38(1.11)	3.76(1.51)	4.48(1.18)	3.21(1.56)	3.35(1.44)	5.83(0.82)	5.89(1.05)	5.98(1.36)
*Czech Republic*	4.11(1.19)	3.50(1.20)	5.12(0.90)	4.56(1.08)	4.39(1.07)	4.45(0.95)	2.83(1.55)	2.88(1.30)	6.01(0.92)	5.67(1.04)	5.95(0.98)
*Germany*	3.59(1.24)	3.19(1.10)	5.07(0.93)	4.13(1.14)	4.44(1.18)	4.27(1.03)	3.60(1.44)	3.01(1.52)	5.93(0.84)	5.48(1.23)	5.04(1.71)
*Spain*	4.37(1.26)	4.21(1.21)	5.35(0.94)	3.45(1.32)	4.29(1.24)	3.76(1.28)	3.21(1.46)	3.91(1.62)	6.25(0.81)	6.15(0.95)	6.55(0.69)
*United Kingdom*	4.38(1.24)	3.80(1.17)	4.76(0.91)	4.60(1.07)	4.93(1.16)	4.29(1.00)	3.34(1.57)	3.61(1.60)	5.82(1.06)	5.66(1.16)	5.06(1.58)
*Hong Kong*	4.71(0.74)	4.18(0.94)	4.69(0.82)	4.72(0.96)	4.91(0.93)	4.58(0.80)	3.75(1.11)	4.10(1.36)	5.23(1.19)	5.13(1.20)	4.57(1.26)
*India*	4.53(1.11)	4.03(1.07)	5.02(0.93)	4.69(0.98)	4.52(1.23)	4.97(1.03)	3.54(1.44)	3.56(1.60)	5.13(1.18)	5.83(1.13)	†
*Italy*	4.82(1.08)	3.65(1.26)	5.53(0.81)	4.03(1.09)	5.12(0.97)	4.72(0.66)	3.57(1.54)	4.48(1.28)	6.07(0.82)	5.91(1.07)	5.14(1.70)
*Japan*	4.55(1.17)	4.06(1.03)	4.46(0.98)	4.54(0.98)	4.83(1.14)	4.06(1.02)	4.18(1.34)	3.89(1.55)	5.10(1.25)	5.34(1.24)	5.21(1.40)
*Kenya*	5.21(1.13)	4.38(1.10)	5.28(1.20)	4.81(1.15)	4.43(1.50)	5.27(1.09)	3.43(1.22)	4.17(1.58)	5.18(1.28)	5.76(1.28)	5.25(1.38)
*South Korea*	4.40(1.35)	3.78(1.08)	4.59(0.90)	4.60(0.89)	4.75(1.05)	4.82(0.88)	3.81(1.36)	3.42(1.44)	5.60(0.80)	5.50(1.23)	5.31(1.80)
*Mexico*	5.06(1.17)	4.19(1.16)	4.76(1.15)	4.98(1.15)	4.19(1.48)	4.56(1.16)	3.41(1.61)	3.93(1.70)	5.57(1.13)	5.18(1.28)	5.25(1.83)
*New Zealand*	4.24(1.12)	3.68(1.15)	4.89(0.92)	4.28(1.11)	4.79(1.31)	4.05(1.02)	3.73(1.55)	3.39(1.59)	5.86(0.83)	5.66(1.12)	6.48(0.65)
*Pakistan*	4.83(1.10)	4.02(1.13)	5.24(0.79)	4.58(1.05)	4.46(1.12)	4.97(0.95)	2.84(1.23)	3.53(1.48)	5.76(1.26)	6.13(0.91)	5.49(1.46)
*Peru*	4.47(1.47)	3.93(1.23)	4.98(1.16)	4.93(1.29)	3.57(1.52)	3.94(1.29)	3.04(1.52)	3.08(1.79)	5.40(1.14)	5.41(1.30)	5.71(1.35)
*Portugal*	4.96(0.99)	4.16(1.17)	4.96(0.97)	4.51(1.19)	4.77(1.13)	4.34(1.12)	3.33(1.53)	3.78(1.37)	6.07(0.90)	5.56(1.19)	4.71(1.98)
*Romania*	4.67(1.27)	3.78(1.25)	5.01(1.18)	4.01(1.39)	4.02(1.23)	4.75(1.06)	3.16(1.58)	2.62(1.40)	5.94(0.98)	5.87(1.26)	5.64(1.44)
*Russia*	4.52(0.82)	4.01(1.10)	4.85(1.00)	4.51(1.21)	4.63(1.11)	4.65(0.86)	3.04(1.56)	3.87(1.64)	5.63(0.68)	5.70(1.09)	6.15(1.17)^†^
*Senegal*	5.48(0.86)	5.05(0.97)	5.53(0.91)	3.04(1.18)	4.44(0.95)	5.81(0.84)	3.37(1.09)	4.35(1.38)	5.41(1.09)	6.39(0.76)	5.22(1.71)
*Thailand*	5.24(0.79)	4.70(0.85)	5.24(0.69)	4.44(0.78)	4.94(0.89)	4.87(0.78)	3.54(1.11)	3.98(1.29)	5.58(0.81)	5.90(1.00)	4.64(1.73)^†^
*Turkey*	4.44(1.17)	4.07(1.22)	4.91(1.06)	4.25(1.29)	4.39(1.33)	4.99(1.04)	3.40(1.36)	3.47(1.56)	5.64(1.00)	5.54(1.19)	4.86(1.76)
*Uganda*	5.52(1.21)	4.94(1.39)	5.33(1.44)	4.04(1.47)	4.65(1.55)	5.37(1.21)	3.33(1.80)	3.76(1.79)	5.74(1.34)	5.75(1.31)	5.83(1.60)
*Ukraine*	3.81(1.09)	3.92(1.03)	5.07(1.02)	4.29(1.12)	4.11(1.15)	4.99(1.04)	3.26(1.30)	3.78(1.34)	5.42(0.84)	5.69(1.11)	4.21(1.81)^†^
*United States*	4.46(1.27)	4.05(1.31)	4.63(1.12)	4.88(1.13)	4.11(1.31)	4.37(1.11)	3.27(1.66)	3.28(1.64)	5.69(1.15)	5.21(1.32)	5.41(1.33)
*Overall*	4.59(1.24)	4.02(1.22)	4.98(1.04)	4.32(1.25)	4.47(1.26)	4.60(1.16)	3.41(1.46)	3.60(1.59)	5.69(1.06)	5.66(1.19)	5.37(1.50)

Fundamental Social Motives are measured on a 7-point Likert scale, and higher numbers indicate greater concern for or importance of the motive. Subscales are Self-protection (SPO), Disease Avoidance (DIS), Affiliation (Group) (AFG), Affiliation (Independence) (AFI), Affiliation (Exclusion Concern) (AFX), Status (STA), Mate Seeking (MAT), Breakup Concern (MRB), Mate Retention (MRT), Kin Care (Family) (KCF), Kin Care (Children) (KCC). Breakup Concern and Mate Retention questions were only answered by participants currently in a relationship. Kin Care (Children) questions were only answered by participants who have children. A version of this table also appears in the Supplementary Materials of a manuscript under review at the time of this publication^32^.

^†^Indicates country samples in which 10 participants or fewer had children/responded to KCC items.

**Table 4:** Fundamental Social Motive means (and standard deviations) by country in Wave 2.

Country/Society	SPO	DIS	AFG	AFI	AFX	STA	MAT	MRB	MRT	KCF	KCC
*Bulgaria*	4.83(1.41)	4.59(1.35)	5.19(1.06)	4.21(1.43)	4.17(1.35)	4.68(1.21)	2.87(1.77)	2.99(1.63)	5.92(0.96)	5.87(1.14)	6.27(1.13)
*Brazil*	5.27(1.19)	5.07(1.14)	5.15(1.06)	4.47(1.22)	4.32(1.44)	4.20(1.19)	2.96(1.53)	3.04(1.50)	5.73(0.98)	5.46(1.27)	6.05(1.21)
*Canada*	4.87(1.27)	4.94(1.28)	4.73(1.12)	4.97(1.16)	4.32(1.36)	4.25(1.16)	2.91(1.62)	2.71(1.56)	5.79(1.12)	5.40(1.37)	5.89(0.96)
*China*	5.21(0.85)	4.57(0.92)	4.74(0.83)	4.52(1.17)	4.97(1.00)	4.84(0.89)	3.74(1.17)	4.25(1.25)	5.78(0.94)	5.59(1.06)	6.18(1.09)
*Colombia*	4.67(1.28)	4.41(1.14)	5.02(0.98)	4.54(1.29)	3.77(1.46)	3.95(1.25)	3.39(1.38)	3.84(1.74)	5.59(0.98)	5.81(1.12)	5.23(1.92)
*Czech Republic*	4.33(1.24)	3.64(1.23)	5.20(1.01)	4.58(1.18)	4.27(1.21)	4.48(1.00)	2.48(1.61)	2.53(1.43)	6.13(0.89)	5.92(1.10)	6.21(1.01)
*Germany*	4.00(1.19)	3.98(1.29)	5.17(0.88)	3.97(1.21)	4.63(1.10)	4.38(1.02)	3.46(1.55)	2.78(1.46)	5.94(1.03)	5.62(1.10)	5.00(1.71)
*Spain*	4.43(1.13)	4.18(1.09)	5.26(0.89)	4.14(1.13)	4.73(1.08)	4.26(1.04)	3.11(1.40)	4.17(1.69)	5.75(1.06)	5.88(1.13)	5.35(1.66)
*United Kingdom*	4.69(1.21)	4.67(1.23)	4.62(1.07)	4.73(1.11)	4.46(1.33)	3.77(1.16)	2.46(1.65)	2.74(1.62)	6.06(0.78)	5.66(1.17)	6.36(0.66)
*India*	4.71(1.17)	4.56(1.17)	5.10(0.99)	4.52(1.16)	4.83(1.22)	5.12(0.97)	4.02(1.53)	3.49(1.76)	5.99(0.71)	5.75(1.07)	5.38(2.14)^†^
*Israel*	4.43(1.07)	4.55(1.14)	5.34(0.89)	3.44(0.96)	4.69(0.98)	5.09(0.85)	4.17(1.63)	2.76(1.33)	6.22(0.70)	6.27(0.80)	4.53(1.96)
*Italy*	5.07(1.02)	4.44(1.27)	5.65(0.79)	4.07(1.18)	4.89(1.12)	4.81(0.69)	3.79(1.56)	4.55(1.35)	6.12(0.81)	6.06(0.87)	6.13(0.99)^†^
*South Korea*	4.95(0.88)	4.95(1.02)	4.47(0.94)	4.56(1.06)	4.52(1.09)	4.54(0.97)	3.20(1.32)	3.03(1.29)	5.38(0.93)	5.39(1.17)	5.68(0.92)
*Lebanon*	4.55(1.45)	4.52(1.39)	4.87(1.09)	4.84(1.35)	4.05(1.42)	4.77(1.12)	3.16(1.82)	2.78(1.54)	5.90(0.86)	5.78(1.24)	6.53(0.43)
*Nigeria*	5.57(1.34)	5.34(1.31)	5.43(1.21)	4.43(1.35)	3.66(1.61)	5.33(1.22)	2.65(1.40)	2.79(1.50)	5.83(1.21)	6.35(0.99)	6.49(0.73)
*Netherlands*	4.17(1.21)	3.94(1.12)	5.09(0.95)	4.24(1.12)	4.69(1.13)	4.11(0.92)	3.37(1.65)	2.96(1.50)	6.07(0.73)	5.86(1.02)	6.08(0.12)^†^
*New Zealand*	4.45(1.06)	4.05(1.07)	5.24(0.75)	4.12(1.23)	5.16(1.02)	4.03(0.91)	3.78(1.53)	3.30(1.48)	6.10(0.74)	5.73(1.09)	6.42(0.82)^†^
*Peru*	4.86(1.24)	4.96(1.18)	5.21(1.06)	4.73(1.24)	3.69(1.39)	4.00(1.24)	2.99(1.35)	3.08(1.65)	5.33(1.30)	5.38(1.34)	5.48(1.52)
*Philippines*	5.18(0.84)	4.57(1.14)	4.98(0.93)	5.38(0.92)	5.01(1.10)	5.12(0.93)	3.75(1.15)	5.08(1.31)	5.19(1.13)	5.54(1.00)	5.77(0.73)
*Russia*	4.40(0.91)	3.99(1.36)	4.80(0.99)	5.13(1.06)	4.36(1.19)	4.62(1.02)	2.66(1.37)	3.45(1.43)	5.50(0.89)	5.59(1.38)	4.72(1.84)
*Saudi Arabia*	5.04(1.35)	4.54(1.03)	4.80(1.36)	4.94(1.57)	4.57(1.74)	4.86(1.21)	3.62(1.25)	4.68(1.85)	4.53(1.51)	5.28(1.28)	5.60(1.23)
*Senegal*	5.53(0.99)	5.28(1.09)	5.60(1.02)	3.12(1.33)	4.33(1.15)	5.78(0.97)	3.38(1.22)	4.04(1.58)	5.40(1.24)	6.43(0.82)	5.30(1.83)
*Singapore*	4.79(1.06)	4.65(1.12)	5.11(0.81)	4.76(1.11)	4.85(1.15)	4.74(0.89)	3.21(1.34)	3.73(1.43)	6.01(0.72)	5.58(1.07)	1.00(0.00)^†^
*Serbia*	4.53(1.14)	4.08(1.35)	4.62(1.18)	4.74(1.12)	4.14(1.36)	4.39(1.01)	3.64(1.62)	2.73(1.79)	6.21(0.85)	5.58(1.21)	6.05(0.82)^†^
*Slovakia*	4.70(1.13)	4.47(1.14)	5.06(0.95)	4.31(1.19)	4.13(1.19)	4.36(0.98)	2.77(1.48)	2.60(1.33)	5.92(0.91)	5.87(1.01)	5.83(1.21)
*Sweden*	4.09(1.20)	4.00(0.98)	5.06(0.85)	4.44(1.34)	4.55(1.38)	4.22(0.95)	3.36(1.25)	2.49(1.50)	4.60(1.12)	4.84(1.03)	5.63(0.93)
*Thailand*	5.16(0.98)	4.72(0.77)	5.20(0.91)	4.75(1.08)	5.12(1.28)	4.73(0.98)	3.56(1.29)	3.80(1.67)	5.56(0.89)	5.48(1.37)	5.02(1.96)
*Turkey*	4.71(1.15)	5.15(1.22)	4.83(1.17)	4.36(1.25)	4.20(1.38)	4.80(1.16)	3.06(1.30)	3.43(1.56)	5.94(0.84)	5.49(1.24)	7.00(0.00)^†^
*United States*	4.97(1.32)	5.13(1.25)	4.43(1.24)	5.17(1.16)	3.63(1.42)	3.70(1.20)	2.62(1.60)	2.16(1.26)	5.90(1.07)	5.40(1.33)	5.83(1.18)
*Overall*	4.79(1.22)	4.61(1.24)	5.05(1.05)	4.40(1.32)	4.44(1.32)	4.56(1.18)	3.23(1.53)	3.23(1.65)	5.73(1.07)	5.70(1.18)	5.76(1.37)

Fundamental Social Motives are measured on a 7-point Likert scale, and higher numbers indicate greater concern for or importance of the motive. Subscales are Self-protection (SPO), Disease Avoidance (DIS), Affiliation (Group) (AFG), Affiliation (Independence) (AFI), Affiliation (Exclusion Concern) (AFX), Status (STA), Mate Seeking (MAT), Breakup Concern (MRB), Mate Retention (MRT), Kin Care (Family) (KCF), Kin Care (Children) (KCC). Breakup Concern and Mate Retention questions were only answered by participants currently in a relationship. Kin Care (Children) questions were only answered by participants who have children. A version of this table also appears in the Supplementary Materials of a manuscript under review at the time of this publication^32^.

^†^Indicates country samples in which 10 participants or fewer had children/responded to KCC items.

#### Life satisfaction

Participants’ life satisfaction was assessed via the Satisfaction with Life Scale (SWLS)^28^ in a subset of countries (see Supplementary Table 2 for SWLS means and standard deviations by country). SWLS was measured in 15 countries in Wave 1 and 28 countries in Wave 2. SWLS was measured in both waves in 10 countries.

#### Basic need fulfilment

In a subset of countries, we also assessed the degree to which participants felt their basic needs (i.e., food availability, water availability, safety, livable temperature/climate, and adequate housing/shelter) were being fulfilled. Basic needs fulfilment was measured in 12 countries in Wave 1 and 28 countries in Wave 2. Basic needs were measured in both waves in 7 countries.

#### Demographic variables

Demographic information on age, gender, relationship status, and number of children was collected in each country. Race/ethnicity was measured using country-appropriate categories as indicated by local collaborators. Participants also indicated where they would place their own subjective socioeconomic status (SES) on a 10-rung subjective social status ladder^29^, in which the lowest rung (1) corresponds to those in society who are worst off in terms of money, education, and respected jobs, and the highest rung (10) corresponds to those who are best off. Supplementary Table 2 provides sample size and participants’ gender, age, and subjective SES by country in each wave.

#### Additional variables

Participants in a small subset of countries were asked additional questions, such as their religion. In Wave 2, participants in some English-speaking countries were asked questions such as how successful they believed themselves to be at accomplishing each of the fundamental social motives. Some were asked how much they would like to know, upon meeting a person for the first time, how important each of the fundamental social motives was to that person.

## Data Records

Datasets^30^ are available as .sav files (for direct use in SPSS) and .csv files on the Open Science Framework (OSF) platform. We provide three types of datasets.

First, we provide a “master” dataset containing sample variables (details below), fundamental social motives, and participant demographics and other individual difference variables for each participant across countries and waves.

Second, we provide “individual country” data files for each sample collected in each of the two waves. Many of these datasets contain additional variables collected in only a subset of countries, or only in one country (e.g., country-specific ethnicity or religion questions, as determined by local collaborators).

Third, we provide a “country-level” data file containing country-level mean values for each fundamental social motive, country-level values for commonly studied cross-cultural variables compiled from published research (e.g., individualism, relational mobility, tightness-looseness), and country-level economic indicators (e.g., GDP and GINI). A complete reference list for these variables is provided in the OSF project^30^.

Due to ethical considerations, raw individual country datasets were cleaned to remove participants who indicated that they were 15-, 16-, or 17-years old (total excluded *N*_under18_ = 81), and to remove potentially identifying information and metadata. Variables to be included in the master dataset (e.g., fundamental motives, gender, age) were renamed and recoded to match the standardized coding of the master dataset and then compiled. Missing data in the fundamental motives items and the Gender, Age, Relationship, N.Children, SubjSES, BirthCountry, and RaceEthnicity variables are indicated by blanks spaces or values of -999, -99, -77, or -66 in various individual country datasets and in the master dataset.

### Sample variables

Sample variables include identifiers for each participant in the master dataset (**masterID**) and for each participant within their individual country dataset (**pID**). Each participant’s country is indicated by the country name (**country.N)**, the country’s three-letter ISO 3166 alpha-3 country code (**ISO3)**, and a numeric code assigned to each country based on ISO3 (**country.ID**). In countries where more than one sample was collected during the same wave, these datasets are distinguished via the **subsample** variable. The wave in which a participant’s data were collected is indicated by the **wave** variable.

We also provide a variable indicating which participants we recommend excluding from analyses (**filter_exclude**, where 1 = *include* and 0 = *exclude*). A second variable (**ExcludeWhy**) indicates the reason why a participant’s data is recommended for exclusion. These reasons include: 1 = *Invalid response on Fundamental Motive item*, 2 = *Invalid response on Age*, 3 = *One or more Fundamental Motive subscale scores (except Mate Retention, Breakup Concern, or Kin Care (Children)) is entirely missing*, 4 = *Other invalid response* (e.g., “9” on a 7-point scale SWLS item). This filter (excluding 2807 cases across all samples) was applied when calculating all descriptive statistics and creating all figures included here.

Each of the above sample variables are included in both the master dataset and the individual country datasets (with the exception of the **masterID** variable, only included in the master dataset).

### Fundamental social motive variables

The eleven Fundamental Social Motive Inventory subscale abbreviations are as follows: Affiliation (Group) = **AFG**, Affiliation (Independence) = **AFI**, Affiliation (Exclusion Concern) = **AFX**, Disease Avoidance = **DIS**, Kin Care (Children) = **KCC**, Kin Care (Family) = **KCF**, Mate Seeking = **MAT**, Breakup Concern = **MRB**, Mate Retention = **MRT**, Self-Protection = **SPO**, and Status = **STA**.

Each Fundamental Social Motive Inventory subscale comprises six items named according to the subscale abbreviation and a number, 1 through 6 (e.g., **AFG1, AFG2**, **AFG3**, etc.). Items are measured from 1 = *Strongly disagree* (indicating low levels of the motive, except for reverse-scored items) to 7 = *Strongly agree* (indicating high levels of the motive, except for reverse-scored items). Certain subscale items need to be reverse-scored for subscale score calculation—these items end in “**R**” and *are not yet reverse-scored* in the datasets (e.g., **AFG4R**).

For each participant, the six items of each subscale were reverse-scored as appropriate and then averaged together to form a subscale score variable. These variables are indicated by fundamental social motive subscale abbreviations followed by no numbers (e.g., **AFG**).

### Individual difference and demographic variables

Demographics and other individual difference variables that were collected in most countries are included in the master dataset. These variables include the participant’s sex (**Gender**, where 1 = *Male*, 2 = *Female*, and, in some datasets 3 = *Other*) and age in years (**Age**). Participants indicated their subjective SES by rating whether they are 10 = *Best off* (in money, education, and respected jobs) to 1 = *Worst off* (**SubjSES**). Participants self-reported their relationship status (**Relationship**), coded 1 = *Single and not currently dating*, 2 = *Single and currently dating*, 3 = *In a committed relationship*, 4 = *Married*, and 5 = *Divorced/Widowed*. Participants self-reported their number of children (**N.Children**) from *0* to *4 or greater*.

Participants in many countries in each wave responded to the five-item Satisfaction with Life scale (**swls1** through **swls5)**, on a 7-point scale with higher scores indicating greater satisfaction with life. These items were then averaged for each participant to form their satisfaction with life score variable (**SWLS)**.

Participants in many countries in each wave indicated whether their basic needs (i.e., enough **food**, enough **water**, a reliable place to **sleep**, a livable **temperature**, and feeling **safe**) were being met, from 1 = *Strongly disagree* to 7 = *Strongly agree*.

Finally, participants in several countries in Wave 2 rated how interested they would be, upon meeting a new person, to learn how important each fundamental social motive was to that person (**learn** variables, each with a corresponding fundamental social motive suffix, e.g., **learnAFG**), from 1 = *Very uninterested* to 7 = *Very interested*.

## Technical Validation

The main instrument used, the Fundamental Social Motives Inventory^14^, as well as the Satisfaction with Life Scale^28^ are published scales with established reliability and validity indicators. These scale and subscale scores were calculated by reverse-scoring items (as appropriate) and averaging subscale items according to the published scale calculation procedures.

English survey materials were translated by native speakers for use across countries. Information regarding the language in which the study was conducted for each sample, as well as information on translation procedures for each language can be found in Supplementary Table 1.

## Usage Notes

This dataset provides numerous opportunities to explore how people’s fundamental social motives vary around the world across two timepoints, as well as to explore factors that may be associated with these cross-cultural variations. By analyzing this dataset alone and in combination with other cross-cultural datasets that include further indicators of culture, values, personality, etc., researchers can explore the following types of scientific questions:

- How do fundamental social motives vary around the world?
- How might ecological variables, such as pathogen prevalence, rates of violence, and income inequality affect people’s fundamental social motives?
- Do demographic variables such as age, gender, number of children, and SES affect people’s fundamental social motives in the same ways around the world?
- How do people’s fundamental social motives relate to cross-cultural dimensions such as individualism/collectivism, relational mobility, and tightness/looseness? How do fundamental social motives relate to values, such as egalitarianism and harmony (Wetzel’s values)?
- How does variation in fundamental social motives around the world relate to variation in other individual difference dimensions, such as Big 5 personality traits?
- How might fundamental social motives predict important societal outcomes, such as a society’s level of innovation or democratic policies?
- How do people’s fundamental social motives affect their happiness around the world^31^ and across time?
- What effect might the COVID-19 pandemic have had on people’s fundamental social motives^32^?

This non-exhaustive list provides examples of the range of important questions this dataset can help us address, to better understand how and why people’s motivations vary across cultures and across time.

### Illustrative exploratory analysis

**Fig. 2:**
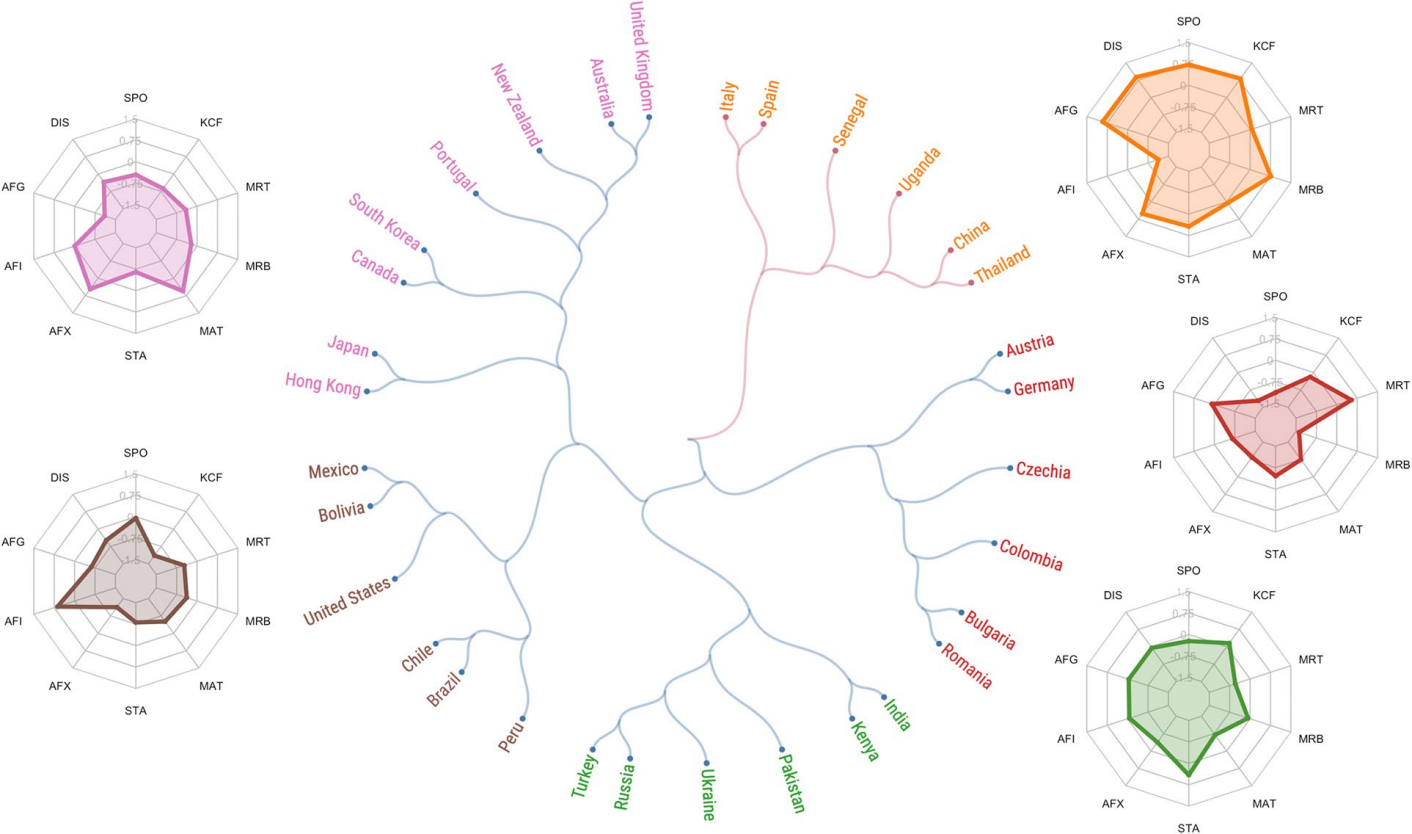
Hierarchical clustering of societies based on fundamental social motives measured in Wave 1. The dendrogram illustrates societies’ similarity on overall fundamental social motive (FSM) profiles (*N* = 8,998 participants) in the Wave 1 data collection. Two countries that branch apart farther from the center are more similar than two countries that branched apart closer to the center. The color of a country’s link represents its membership to a main cluster (two clusters: red and blue), whereas the color of its name represents its membership to a sub-cluster (five sub-clusters). The radar chart next to each cluster of the dendrogram shows average *z*-scores of each FSM subscale for all countries in that cluster. Subscales are Self-Protection (SPO), Disease Avoidance (DIS), Affiliation (Group) (AFG), Affiliation (Independence) (AFI), Affiliation (Exclusion Concern) (AFX), Status (STA), Mate Seeking (MAT), Breakup Concern (MRB), Mate Retention (MRT), Kin Care (Family) (KCF).

**Fig. 3:**
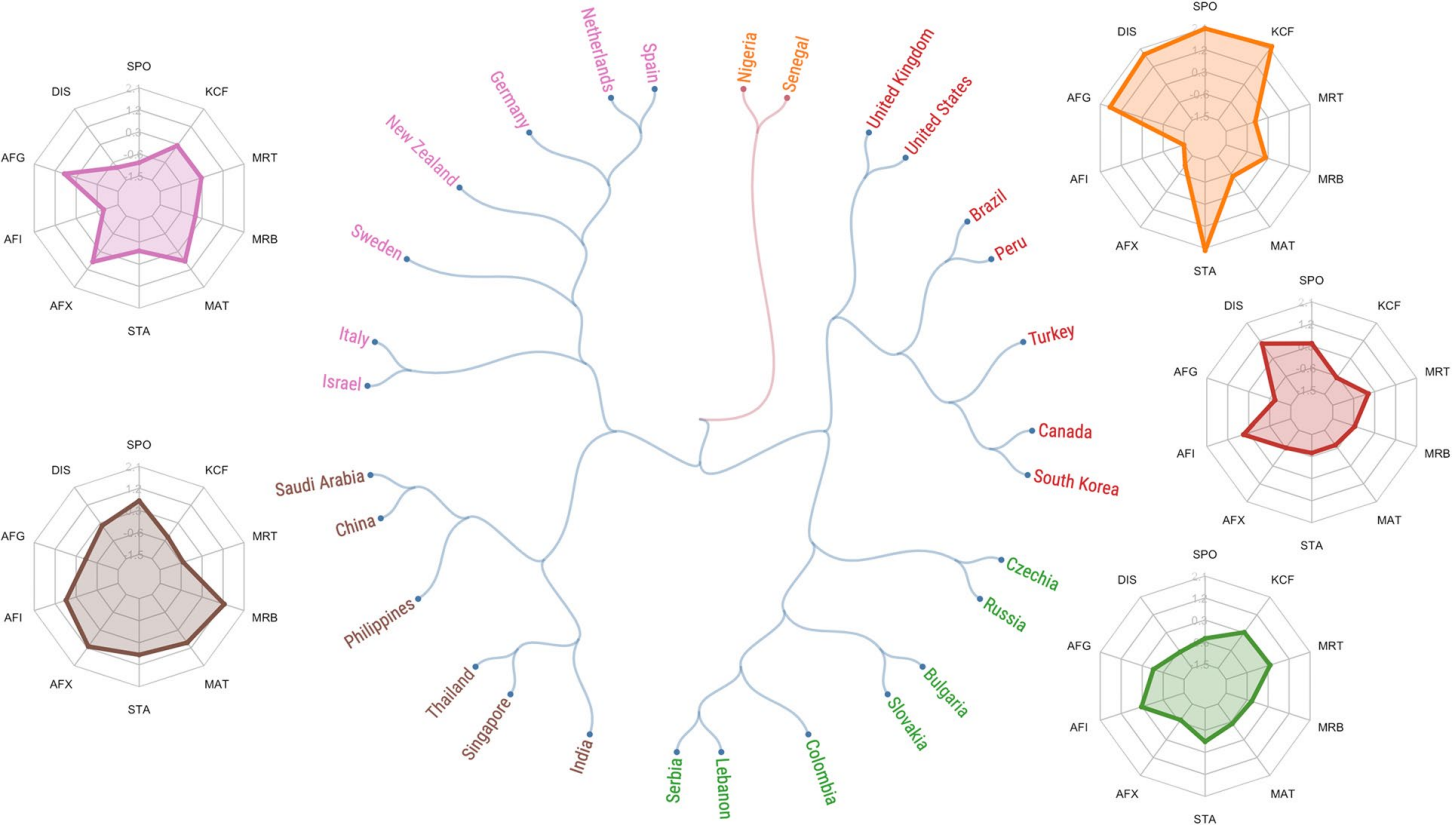
Hierarchical clustering of societies based on fundamental social motives measured in Wave 2. The dendrogram illustrates societies’ similarity on overall fundamental social motive (FSM) profiles (*N* = 6,917 participants) in the Wave 2 data collection. Two countries that branch apart farther from the center are more similar than two countries that branched apart closer to the center. The color of a country’s link represents its membership to a main cluster (two clusters: red and blue), whereas the color of its name represents its membership to a sub-cluster (five sub-clusters). The radar chart next to each cluster of the dendrogram shows average *z*-scores of each FSM subscale for all countries in that cluster. Subscales are Self-Protection (SPO), Disease Avoidance (DIS), Affiliation (Group) (AFG), Affiliation (Independence) (AFI), Affiliation (Exclusion Concern) (AFX), Status (STA), Mate Seeking (MAT), Breakup Concern (MRB), Mate Retention (MRT), Kin Care (Family) (KCF).

To help illustrate the potential of the dataset, we present a straightforward exploration of the variation in fundamental social motives around the world in each wave. One way to think about cross-societal similarities and differences is to consider how different societies cluster, based on similarities and differences in their overall fundamental social motive profiles. Figure 2 presents a dendrogram of Wave 1 countries, and Fig. 3 presents a dendrogram of Wave 2 countries. In each dendrogram, countries branch into two main clusters and five subclusters, based on similarity of motive profiles (indicated by different colors). The average motive profile of each subcluster is illustrated by a radar chart.

In Fig. 2, Wave 1 countries branch into two main clusters and five subclusters. These branches reveal that countries do not cluster into traditional West vs. Rest or Rich vs. Poor clusters. Yet, the clusters are hardly arbitrary. For example, New Zealand, Australia, Canada, and the United Kingdom are on one branch. Austria is closest to Germany, as is Spain to Italy. Likewise, Bolivia, Mexico, Brazil, Chile, and Peru are in the same subcluster. However, clusters also deviate from previous categorizations of the world’s cultures. For example, most English-speaking countries cluster with wealthy East Asian democracies. The United States, though, clusters with several Latin American countries. Further, Senegal and Uganda form a subcluster with Italy, Spain, Thailand, and China. These results suggest that fundamental social motives not only capture sensible patterns of cultural clustering that have previously been posited, but also reveal new and sometimes surprising similarities between societies (e.g., between South Korea and Canada, between the United States and Peru).

In Fig. 3, Wave 2 (mid-pandemic) countries branch in two main clusters and five subclusters. These branches reveal some familiar patterns. For example, all post-communist societies belong to one subcluster, all but one West European country belong to one subcluster, and the two East African societies in Wave 2 cluster closest to each other. The clusters also reveal some surprising patterns, however. For example, Colombia and Lebanon cluster with post-communist European societies, and South Korea again clusters closest to Canada.

#### Dendrograms

Separately for each wave (across the 32 societies in the Wave 1 sample, and across the 29 societies in the Wave 2 sample), we standardized each of ten fundamental social motive subscales (excluding the Kin Care (Children) subscale because 10 participants or fewer completed this scale in several countries; these countries are indicated in Tables 3 and 4). We then utilized a Python implementation^33^ of hierarchical agglomerative clustering (HAC), using Euclidean distance and Ward variance minimization linkage criterion^34,35^ to create each dendrogram.

## Supplementary information

Fundamental social motives measured across forty-two cultures in two waves: Supplementary Information

## Data Availability

All code used to process and visualize the data, including information on software packages used, is freely available in the OSF project^30^.
