# Publisher Correction: Fundamental social motives measured across forty-two cultures in two waves

**DOI:** 10.1038/s41597-022-01672-0

**Published:** 2022-09-20

**Authors:** Cari M. Pick, Ahra Ko, Douglas T. Kenrick, Adi Wiezel, Alexandra S. Wormley, Edmond Awad, Laith Al-Shawaf, Oumar Barry, Yoella Bereby-Meyer, Watcharaporn Boonyasiriwat, Eduard Brandstätter, Suzan Ceylan-Batur, Bryan K. C. Choy, Ana Carla Crispim, Julio Eduardo Cruz, Daniel David, Oana A. David, Renata Pereira Defelipe, Pinar Elmas, Agustín Espinosa, Ana Maria Fernandez, Velichko H. Fetvadjiev, Stefka Fetvadjieva, Ronald Fischer, Silvia Galdi, Oscar Javier Galindo-Caballero, Elena V. Golovina, Galina M. Golovina, Luis Gomez-Jacinto, Sylvie Graf, Igor Grossmann, Pelin Gul, Peter Halama, Takeshi Hamamura, Shihui Han, Lina S. Hansson, Hidefumi Hitokoto, Martina Hřebíčková, Darinka Ilic, Jennifer Lee Johnson, Mane Kara-Yakoubian, Johannes A. Karl, Jinseok P. Kim, Michal Kohút, Julie Lasselin, Hwaryung Lee, Norman P. Li, Anthonieta Looman Mafra, Oksana Malanchuk, Simone Moran, Asuka Murata, Jinkyung Na, Serigne Abdou Lahat Ndiaye, Jiaqing O, Ike E. Onyishi, Eddieson Pasay-an, Muhammed Rizwan, Eric Roth, Sergio Salgado, Elena S. Samoylenko, Tatyana N. Savchenko, Catarina Sette, A. Timur Sevincer, Eric Skoog, Adrian Stanciu, Eunkook M. Suh, Daniel Sznycer, Thomas Talhelm, Fabian O. Ugwu, Ayse K. Uskul, Irem Uz, Jaroslava Varella Valentova, Marco Antonio Correa Varella, Liuqing Wei, Danilo Zambrano, Michael E. W. Varnum

**Affiliations:** 1 Department of Psychology, Arizona State University, Tempe, AZ 85287 USA; 2 Office of the Chief Scientist, Environmental Defense Fund, New York, NY 10010 USA; 3 Department of Economics, University of Exeter Business School, Exeter EX4 4PU, England, UK; 4 Department of Psychology, University of Colorado, Colorado Springs, CO 80309 USA; 5 Department of Psychology, University Cheikh Anta Diop of Dakar (UCAD), Dakar, 10700 Senegal; 6 Department of Psychology, Ben-Gurion University of the Negev, Beer-Sheva, 84105 Israel; 7 Faculty of Psychology, Chulalongkorn University, Bangkok, 10330 Thailand; 8 Department of Economic Psychology, Johannes Kepler University Linz, 4040 Linz, Austria; 9 Department of Psychology, TOBB University of Economics and Technology, 06510 Ankara, Turkey; 10 School of Social Sciences, Singapore Management University, Singapore, 188065 Singapore; 11 eduLab21, Ayrton Senna Institute, São Paulo, 05423-040 Brazil; 12 Department of Psychology, Universidad de los Andes, Bogotá, Cundinamarca Colombia; 13 Department of Clinical Psychology and Psychotherapy, Babeş-Bolyai University, Cluj-Napoca, 400347 Romania; 14 Department of Experimental Psychology, Institute of Psychology, University of São Paulo, São Paulo, 05508-030 Brazil; 15 Department of Psychology, Adnan Menderes University, 09010 Aydın, Turkey; 16 Grupo de Psicología Política y Social (GPPS), Departamento de Psicología, Pontificia Universidad Católica del Perú, San Miguel, 15088 Lima, Peru; 17 School of Psychology, University of Santiago, Santiago, Estación Central, Región Metropolitana Chile; 18 Department of Psychology, University of Amsterdam, 1018 WS Amsterdam, Netherlands; 19 WorkWell Research Unit, North-West University, Potchefstroom, 2520 South Africa; 20 Department of Bulgarian Language, Sofia University, Sofia, Bulgaria; 21 School of Psychology, Victoria University of Wellington, Wellington, 6012 New Zealand; 22 Instituto D’Or de Pesquisa e Ensino, Rio de Janeiro, 22281-100 Brazil; 23 Department of Psychology, University of Campania Luigi Vanvitelli, 81100 Caserta, Italy; 24 Faculty of Education, Human and Social Sciences, Universidad Manuela Beltran, Bogotá, Colombia; 25 Institute of Psychology Russian Academy of Science, Moscow, 129366 Russia; 26 Department of Social Psychology, Social Work and Social Anthropology, University of Málaga, 29016 Málaga, Spain; 27 Institute of Psychology, Czech Academy of Sciences, 110 00 Nové Město, Prague, Czechia; 28 Department of Psychology, University of Waterloo, Waterloo, Ontario N2L 3G1 Canada; 29 Department of Sustainable Health (Campus Fryslân), University of Groningen, 8911CE Leeuwarden, Netherlands; 30 Center of Social and Psychological Sciences, Slovak Academy of Sciences, 841 04, Bratislava, Slovakia; 31 School of Psychology, Curtin University, Bentley, WA 6102 Perth, Australia; 32 School of Psychological and Cognitive Sciences, Peking University, Beijing, 100871 China; 33 Stress Research Institute, Department of Psychology, Stockholm University, 106 91 Stockholm, Sweden; 34 Division of Psychology, Department of Clinical Neuroscience, Karolinska Institutet, 171 77 Solna, Sweden; 35 Osher Center for Integrative Medicine, ME Neuroradiologi, Karolinska Universitetssjukhuset, 171 77 Solna, Sweden; 36 School & Graduate School of Humanities, Kwansei Gakuin University, Nishinomiya, Hyogo 662-8501 Japan; 37 Department of Psychology, Faculty of Philosophy, University of Niš, Niš, 18000 Serbia; 38 Department of Community Sustainability, Michigan State University, East Lansing, MI 48824 USA; 39 Department of Psychology, Toronto Metropolitan University, Toronto, Ontario M5B 2K3 Canada; 40 School of Psychology, Dublin City University, Dublin, 9 Ireland; 41 Department of Psychology, Yonsei University, Seoul, 03722 South Korea; 42 Faculty of Philosophy and Arts, University of Trnava, 917 01 Trnava, Slovakia; 43 Institute for Social Research, University of Michigan, Ann Arbor, MI 48104 USA; 44 Department of Management, Ben-Gurion University of the Negev, Beer-Sheva, 84105 Israel; 45 Graduate School of Letters, Hokkaido University, Sapporo, Hokkaido 060-0810 Japan; 46 Department of Psychology, Sogang University, Seoul, 04107 South Korea; 47 Department of Sociology, University Cheikh Anta Diop of Dakar (UCAD), Dakar, 10700 Senegal; 48 Department of Psychology, Aberystwyth University, Aberystwyth, SY23 3UX Wales UK; 49 Department of Psychology, University of Nigeria, Nsukka, Nigeria; 50 College of Nursing, University of Hail, Hail, 55476 Saudi Arabia; 51 Department of Psychology, University of Haripur, Haripur, 22620 Khyber Pakhtunkhwa Pakistan; 52 Experimental Research Unit (ERU), Department of Psychology, Universidad Católica Boliviana, La Paz, Bolivia; 53 Department of Management and Economics, Universidad de La Frontera, Temuco, Araucanía Chile; 54 Department of Psychology, University of Hamburg, 20146 Hamburg, Germany; 55 Department of Peace and Conflict Research, Uppsala University, 753 20 Uppsala, Sweden; 56 Department of Monitoring Society and Social Change, Gesis-Leibniz Institute for the Social Sciences, 68072 Mannheim, Germany; 57 Department of Psychology, Oklahoma State University, Stillwater, OK 74078 USA; 58 Behavioral Science, University of Chicago, Chicago, IL 60637 USA; 59 Department of Psychology, Alex Ekwueme Federal University, Ndufu-Alike, Ebonyi State Nigeria; 60 School of Psychology, University of Kent, Canterbury, CT2 7NP UK; 61 Department of Education, Hubei University, Wuhan, Hubei 430061 China; 62 Department of Psychology, Fundación Universitaria Konrad Lorenz, Bogotá, Colombia

**Keywords:** Human behaviour, Anthropology

Correction to: *Scientific Data* 10.1038/s41597-022-01579-w, published online 16 August 2022

In the html version of this article the affiliation details for Marco Antonio Correa Varella were incorrectly given as ‘eduLab21, Ayrton Senna Institute, São Paulo, 05423-040, Brazil’, but should have been ‘Department of Experimental Psychology, Institute of Psychology, University of São Paulo, São Paulo, 05508-030, Brazil’. This has now been corrected in the HTML version of the Article. The PDF version of the Article was correct at the time of publication.

