# Supplementary material for: Fundamental social motives measured across forty-two cultures in two waves
Source: Sci Data. 2022 Aug 16;9:499. doi: 10.1038/s41597-022-01579-w (PMC9380674; doi:10.1038/s41597-022-01579-w)
Supplement: Supplementary file 1 — Fundamental social motives measured across forty-two cultures in two waves: Supplementary Information [file 41597_2022_1579_MOESM1_ESM.pdf]

Fundamental social motives measured across forty-two cultures in two waves

Supplementary Information

Table of Contents

Page

Supplementary Table 1. Data collection details by country……………………………………………………………………….. 2

Supplementary Table 2. Sample size, age, subjective SES, and life satisfaction by country in each wave… 6

Country/ Society	*Subsample*	*Region*	*Sample Type*	*Wave 1*	*Wave 2*	*Survey Language*	*Translation Procedure*
Australia		Perth	University	June 2016–Apr. 2017	-	English	-
Austria		Linz	University	April 2016–June 2016	-	German	Translated by native speaker; back-translated by 2nd native speaker
Bolivia		La Paz	University	2nd semester of 2018	-	Spanish	Used Spanish survey[a]
Brazil	1	São Paulo	University, Community[b]	Sept. 2018–Oct. 2018	Apr 2020–June 2020	Portuguese	Translated by 2 native speakers; back-translated by 3rd native speaker
	2		Community[c]	-	Apr. 2020–Nov. 2020		Translated by 2 native speakers; checked by 3rd native speaker
Bulgaria		Sofia	University, Community[d]	June 2016–Nov. 2016	May 2020	Bulgarian	Translated by native speaker; checked by 2nd native speaker
Canada	1	Waterloo	University	2nd semester of 2016	-	English	-
			Online	-	May 2020		
	2		Online	-	Apr. 2020		
Chile	1	Santiago, various cities	University, Community[e]	Oct. 2018–Dec. 2018	-	Spanish	Used Spanish survey[a]
	2	Temuco	University	Oct. 2018–Apr. 2019	-		Used Spanish survey[a]; language adjusted by 2 native speakers
China		Beijing	University, Online	Oct. 2017	-	Chinese	Translated by native speaker
		Wuhan	University	-	May 2020		Translated by native speaker; checked by English speaker
Colombia		Bogotá, various cities	University, Community[c]	Dec. 2017–Jan. 2018	Apr. 2020–May 2020	Spanish	Translated by native speaker; back-translated by English speaker
Czech Republic			University, Community[f]	Nov. 2018–Dec. 2018	May 2020–June 2020	Czech	Translated by 2 native speakers; checked by 3rd native speaker
Germany	1	Hamburg	University	Nov. 2017–Dec. 2017	Apr. 2020–June 2020	German	Translated by native speaker; back-translated by 2nd native speaker
	2		Community				
Hong Kong		Shatin	University	June 2016	-	Chinese	Used China team's materials; language adjusted by native speaker
India		Chandigarh	University	May 2019	May 2020–June 2020	English	-
Israel		Beer-Sheva	University	-	May 2020	Hebrew	Translated by native speaker; checked by 2nd native speaker

Italy		Padova	University	Oct. 2016–Nov. 2016	-	Italian	Translated by native speaker; checked by 2nd native speaker
		Caserta	University	-	Apr. 2020		
Japan	1	Hokkaido	University	2016–2017	-	Japanese	Translated by native speaker; checked by 2nd native speaker
	2	Kyoto	University	2016	-		Translated by native speaker; checked by 2nd native speaker
Kenya		Nairobi	University, Community	Oct. 2018–Oct. 2019	-	English	-
Lebanon			Community[g]	-	May 2020–Sep. 2020	English	-
Mexico			Online	Nov. 2019	-	Spanish	Used Spanish survey[a]
Netherlands		Twente, Groningen	University, Community[h]	-	May 2020–Oct. 2020	English	-
New Zealand		Wellington	University	Aug. 2017–Sept. 2017	Apr. 2020	English	-
Nigeria		Nsukka	University[i]	-	Apr. 2020–June 2020	English	-
Pakistan		Islamabad, Karachi, Lahore, Peshawar	University, Community	July 2016–Sept. 2016	-	English	-
Peru		Lima	University, Online	Apr. 2019	May 2020–Sept. 2020	Spanish	Used Spanish survey[a]; language adjusted by native speaker
Philippines		Cordillera Admin. Region	Community	-	Apr. 2020–May 2020	English	-
Portugal			Online	Nov. 2019	-	Portuguese	Used Brazil team's survey materials
Romania		Cluj-Napoca	University	June 2016–Oct. 2016	-	Romanian	Translated by native speaker; back-translated by 2nd native speaker; checked by 3rd native speaker
Russia			University, Community, Online	Late 2018–Feb. 2019	Nov. 2020–May 2021	Russian	Translated and back-translated by 4 native speakers
Saudi Arabia		Hail	Community	-	Apr. 2020–May 2020	English	-
Senegal		Dakar	University, Community	Mar. 2019–Aug. 2019	Apr. 2020–July 2020	French	Translated by native speaker
Serbia		Belgrade, Nis, Zajecar	University, Community[j]	-	May 2020–Sept. 2020	Serbian	Translated by 2 native speakers

Singapore			University	-	May 2020	English	-
Slovakia			Online	-	May 2020	Slovak	Translated by 2 native speakers
South Korea	1	Seoul	University	Oct. 2016	-	Korean	Translated by 2 native speakers; checked by multiple native speakers
	2	Seoul	University	June 2019–Oct. 2019	-		
		Yonsei	Online	-	May 2020		
Spain		Málaga	Community	2017-2018	-	Spanish	Translated by native speaker, back-translated by native speaker
		Málaga	University	-	Apr. 2020–June 2020		
Sweden	1	Uppsala	University	-	May 2020–June 2020	English or Swedish[k]	Translated by native speaker; checked by 2nd native speaker
	2	Stockholm, various cities	University, Community[c]	-	May 2020–Oct. 2020	Swedish	Translated by native speaker; checked by 2nd native speaker
Thailand		Bangkok	University	Jan. 2019–Feb. 2019	Sept. 2020–Oct. 2020	Thai	Translated by native speaker; back-translated by a translation center; checked by 2nd native speaker
Turkey	1	Ankara	University	Mar. 2016	-	Turkish	Translated by native speaker; back-translated by 2 other native speakers
	2	Istanbul, Izmir, Aydin, various cities	University, Community[h]	Apr. 2016–Nov. 2016	May 2020		
Uganda		Waskio, Kampala Districts	University, Community	2016	-	English[l]	Items translated by native speaker for participants as needed[l]
Ukraine		Lviv	University	June 2016	-	Ukrainian	Translated by native speaker; back-translated by 2nd native speaker
United Kingdom	1	Kent	University	May 2016–Nov. 2016	-	English	-
			Online	-	May 2020		
	2	Wales	University[i]	Oct. 2018–Feb. 2019	-		
			Online	-	May 2020		
United States	1	Arizona	University	Sept. 2016–Oct. 2016	-	English	-
	2		Online	Jan. 2018	June 2020		
	3		Community[m]	Mar. 2016–Sept. 2016	-		

Supplementary Table 1. Data collection details by country. For each data collection, the Sample Type indicates whether participants were from a university sample, community sample, and/or online paid workforce (e.g., Prolific, Amazon's Mechanical Turk); where available, community sample recruitment procedures or additional Sample Type details are included via table notes. The city/cities or Region of the country where data collection took place, if applicable, is described. Multiple data collections that occurred within a single country in a single wave are distinguished via the Subsample numbers, which correspond to the subsample variable in the master dataset and individual country dataset

files. Approximate start and end dates of data collection for each sample are indicated, by wave. The Survey Language indicates the language in which survey materials were administered, and the Translation Procedure describes the process of translation and back-translation and/or translation checking for all non-English materials. In the Translation Procedure, "native speaker" refers to a multilingual individual who is a native speaker of the "Survey Language," and "English speaker" refers to a multilingual individual who is a native speaker of English and uses the "Survey Language" as an additional language.

[a]The survey materials were translated to Spanish by a native speaker and back-translated by an English speaker. These materials were offered to research teams for use (and for adaptation, as deemed appropriate) in Spanish-speaking countries.

[b]Recruited via newsletter from the university, which is open to any member of the public.

[c]Recruited via social media.

[d]Recruited via researchers' personal contacts and snowball sampling.

[e]Students from multiple universities and community members were recruited via lab database and social networks.

[f]Recruited via email network of former university students and via institution's social media.

[g]Recruited via social media and email lists.

[h]Recruited via social media, social networks, snowball sampling.

[i]Sample includes university students and staff.

[j]Recruited via social networks.

[k]Participants chose whether to participate in English or Swedish.

[l]Survey materials were presented in English but if respondent did not understand a question, Research Assistants translated the question into Luganda, Acholi, or Lugbara.

[m]Recruited via blog post.

	Wave 1					Wave 2				
	N		Subjective SES	Age	SWLS	*N*		Subjective SES	Age	SWLS
**Country/Society**	*Total*	*Female (Other/Missing)*	*Mean (SD)*	*Mean (SD)*	*Mean (SD)*	*Total*	*Female (Other/Missing)*	*Mean (SD)*	*Mean (SD)*	*Mean (SD)*
Australia	176	138	6.35 (1.43)	22.97 (6.86)						
Austria	204	133 (8)	6.80 (1.34)	24.34 (4.84)						
Bolivia	172	109	7.31 (1.24)	21.16 (2.65)	4.74 (1.18)					
Brazil	208	159	6.91 (1.55)	31.62 (10.66)	4.51 (1.34)	278	187 (14)	6.83 (1.69)	33.03 (10.67)	4.53 (1.39)
Bulgaria	200	158 (9)	5.66 (1.43)	21.85 (3.77)		221	142 (25)	4.59 (1.53)	30.08 (11.53)	4.44 (1.34)
Canada	241	152 (8)		20.27 (2.24)		416	204 (15)	6.22 (1.67)	31.72 (10.89)	4.39 (1.38)
Chile	261	156 (5)	5.88 (1.48)	22.75 (5.08)	4.71 (1.24)					
China	200	124	5.83 (1.64)	22.86 (2.61)		277	165 (26)	5.51 (1.67)	22.35 (4.99)	3.54 (1.24)
Colombia	181	126	6.49 (1.69)	26.55 (9.78)		267	188 (12)	6.59 (1.61)	24.61 (8.59)	4.67 (1.26)
Czech Republic	506	362	6.99 (1.47)	32.25 (7.29)	5.00 (1.18)	215	137 (5)	7.08 (1.29)	35.47 (6.53)	5.23 (1.15)
Germany	221	165 (1)	6.78 (1.43)	25.30 (6.27)		218	182 (5)	7.10 (1.39)	25.22 (6.28)	5.02 (1.24)
Hong Kong	242	167 (1)	5.65 (1.57)	24.80 (7.13)						
India	169	106	6.70 (1.64)	20.88 (2.66)	3.92 (1.07)	230	49 (51)	6.62 (1.66)	20.31 (3.84)	3.78 (1.19)
Israel						234	201 (1)	6.78 (1.19)	24.75 (1.49)	5.06 (1.01)
Italy	205	144	6.40 (1.38)	21.81 (3.55)		271	130	6.46 (1.38)	21.80 (3.86)	4.41 (1.25)
Japan	620	303 (10)	5.57 (1.67)	19.62 (1.33)						
Kenya	399	198 (14)	5.49 (1.82)	23.88 (4.87)	4.02 (1.31)					
Lebanon						153	71 (50)	6.52 (1.80)	29.45 (7.14)	3.78 (1.41)
Mexico	196	66 (1)	6.62 (1.56)	28.21 (7.99)	4.12 (1.45)					
Netherlands						233	157 (16)	7.18 (1.26)	20.74 (2.65)	4.86 (1.18)
New Zealand	344	265 (3)	6.22 (1.42)	20.65 (5.53)		202	157	6.32 (1.48)	19.31 (2.96)	
Nigeria						87	20 (27)	6.04 (2.41)	37.66 (9.69)	4.33 (1.49)
Pakistan	119	69 (5)	6.44 (1.70)	28.97 (8.89)						
Peru	128	78 (10)	6.49 (1.70)	38.36 (18.98)	4.14 (1.37)	179	122 (14)	6.52 (1.68)	39.1 (19.26)	4.18 (1.19)
Philippines						215	111 (28)	6.21 (1.43)	29.62 (6.94)	4.92 (0.97)
Portugal	201	66 (1)	6.50 (1.35)	24.35 (6.23)	4.14 (1.35)					
Romania	217	179 (3)	6.59 (1.24)	24.09 (6.87)						
Russia	84	60	5.89 (1.72)	20.58 (5.84)	4.57 (1.31)	112	94	5.44 (1.69)	29.72 (12.09)	4.86 (1.25)
Saudi Arabia						81	29 (25)	7.42 (2.25)	33.84 (10.59)	4.89 (1.88)
Senegal	769	356 (8)	5.29 (1.93)	22.92 (2.22)	4.05 (1.16)	612	335 (43)	5.00 (1.90)	22.55 (2.35)	3.83 (1.26)
Serbia						67	58 (2)	6.09 (1.52)	23.89 (6.1)	4.50 (1.23)

Singapore						249	171 (4)	6.33 (1.45)	22.57 (1.77)	4.45 (1.19)
Slovakia						219	107 (1)	6.13 (1.58)	39.87 (11.7)	4.18 (1.37)
South Korea	311	207 (2)	6.04 (1.62)	21.25 (2.21)	4.69 (1.21)	213	106 (1)	5.37 (1.77)	39.92 (10.33)	3.73 (1.32)
Spain	431	222		23.58 (3.56)		165	136 (10)	6.56 (1.62)		4.47 (1.31)
Sweden						379	273 (5)	6.55 (1.73)	28.19 (9.67)	4.73 (1.37)
Thailand	173	122 (6)	6.03 (1.30)	19.78 (3.12)	4.18 (0.92)	123	94 (4)	5.50 (1.48)	19.66 (2.72)	3.92 (1.33)
Turkey	435	273 (5)	6.67 (1.45)	22.83 (5.83)		291	170 (54)	5.62 (1.56)	21.81 (3.34)	3.68 (1.30)
Uganda	90	36	4.78 (2.83)	27.20 (6.35)						
Ukraine	194	140	6.31 (1.55)	19.94 (1.36)						
United Kingdom	492	408 (4)	6.21 (1.49)	23.51 (9.99)	4.46 (1.46)	405	269 (6)	5.44 (1.62)	33.60 (11.87)	4.14 (1.36)
United States	609	338 (7)	6.37 (1.70)	32.25 (12.55)	4.50 (1.59)	305	153 (5)	5.31 (1.80)	45.37 (15.84)	4.02 (1.61)

Supplementary Table 2. Sample size, age, subjective SES, and life satisfaction by country in each wave. Demographic information for each sample collected before or during the pandemic, by country. The number of participants in each country who identified as female is indicated, with the number of participants who marked "other" or declined to answer indicated in parentheses. For subjective socioeconomic status (SES), 10 indicates the participant believes that, in their country, they are among the "best off" in terms of money, education, and respected jobs, and 1 indicates "worst off." Satisfaction with Life Scale was measured on a 7-point scale, with higher scores indicating more satisfaction with life. A version of this table also appears in the Supplementary Materials of a manuscript under review at the time of this publication[32].
